# The rates of vaginal births after cesarean section have increased during the last decades: a nationwide register-based cohort study in Finland

**DOI:** 10.1007/s00404-023-07010-y

**Published:** 2023-04-05

**Authors:** Matias Vaajala, Rasmus Liukkonen, Ville Ponkilainen, Maiju Kekki, Ville M. Mattila, Ilari Kuitunen

**Affiliations:** 1 Faculty of Medicine and Life Sciences, University of Tampere, Tampere, Finland; 2 Department of Surgery, Central Finland Central Hospital Nova, Jyväskylä, Finland; 3 Department of Obstetrics and Gynecology, Tampere University Hospital, Tampere, Finland; 4 Center for Child, Adolescent and Maternal Health Research, Faculty of Medicine and Health Technology, Tampere University, Tampere, Finland; 5 Department of Orthopaedics and Traumatology, Tampere University Hospital, Tampere, Finland; 6 Department of Pediatrics, Mikkeli Central Hospital, Mikkeli, Finland; 7 Institute of Clinical Medicine and Department of Pediatrics, University of Eastern Finland, Kuopio, Finland

**Keywords:** Cesarean section, Trial of labor after cesarean section, Epidemiology, Obstetrics

## Abstract

**Purpose:**

Epidemiological studies assessing the effects of previous cesarean section (CS) on subsequent delivery mode using large nationwide study populations. This study aims to calculate the incidence rates of trial of labors after cesarean section (TOLACs) and evaluate the annual rates of vaginal births after cesarean section (VBAC) during the last decades in Finland.

**Methods:**

Data from the National Medical Birth Register (MBR) were used to evaluate incidence rates of VABC in the Finnish population (1998–2018). All nulliparous women having their first and second pregnancy during our study period, and with the mode of delivery identified in both of these pregnancies were included in this study. Absolute annual numbers and incidence rates for TOLACs, elective CS, and VBAC were calculated.

**Results:**

The absolute number of TOLACs had an increasing trend during our study period, increasing up to 2118 TOLACs in 2016. The incidence rates for elective CS after the first CS had a decreasing trend, decreasing from 45% in 1999, to 28% in 2018. The absolute number of VBACs had an increasing trend during our study period, peaking in 2016 (1466 VBACs). The rates for VBAC remained relatively constant, ranging between 38 and 52%, but a slightly increasing trend at the end of the study period was seen.

**Conclusion:**

Despite the increasing annual total number of deliveries with CS in the first pregnancy, the absolute numbers and rates for VBACs have increased towards the end of the study period in Finland. The epidemiology of TOLACs and VBACs should be better studied around the world, as with the rapidly increasing rate of CSs, these events are becoming more common challenges in health care.

## What does this study add to the clinical work

**Table:** 

This study adds information about the epidemiology of vaginal births after cesarean section, and repat cesarean sections and trial of labor after cesarean sections, which are poorly studied, especially in Finland.	
Despite the increasing annual total number of deliveries with cesarean section in the first pregnancy, the absolute numbers, and rates for trials of labors after caesarean section have increased during last decades in Finland.	

## Introduction

Vaginal births after cesarean section (VBACs) are an alternative to repeated cesarean sections (CSs), as multiple repeat CS are known to be risk factors for adverse events, such as uterine rupture and intraoperative complications [1]. The trend of increasing cesarean section (CS) rates had evoked worldwide attention for both healthcare workers and the general population. Many studies have assessed the worldwide incidence of CS, and it has been found to be increasing rapidly [2, 3]. However, according to a large nationwide study in China, the incidence of VBACs has also had an increasing trend during the last decade [4].

Despite the rapidly increasing incidence globally, the rates of CS have remained low in Finland. According to the Finnish institute for health and welfare (THL), [5] the overall proportion of CS during the last decades in Finland was approximately 16%. Despite these findings, the trends in the incidence of trials of labor after cesarean section (TOLACs) and VABCs are not thoroughly studied in Finland.

It is known, that multiple cesarean deliveries are associated with more difficult surgery and increased blood loss compared with a second planned cesarean delivery and that the risk of major complications increases with a cesarean delivery number [6]. According to a 10-year survey in the United States, the rates of successful VBAC in the United States are between 38.5 and 69.8% [7]. A large study in Taiwan found out that the success rate of VBAC among those mothers who ended up attempting vaginal delivery was 85% [8].

Even though the risks and advances of VBACs are well studied, epidemiological studies and studies assessing the effects of previous CS on subsequent delivery mode using large nationwide study populations, especially in Finland are lacking. This study aims to calculate the incidence rates of trial of labors after cesarean section (TOLACs) and evaluate the annual rates of vaginal births after cesarean section (VBAC) during the last decades in Finland.

## Materials and methods

In this nationwide retrospective register-based cohort study, data from the National Medical Birth Register (MBR) were used to evaluate incidence rates of VABC in the Finnish population. The MBR is maintained by the Finnish Institute for Health and Welfare (THL). The study period was from 1 January 1998 to 31 December 31, 2018.

The MBR contains data on pregnancies, delivery statistics, and the perinatal outcomes of all births with a birthweight of  ≥  500 g or a gestational age of ≥  22^+0^ weeks. The MBR has high coverage and quality (the current coverage is nearly 100%) [9, 10]. All nulliparous women, who had their first and second pregnancy during our study period, and with the mode of delivery identified in both of these pregnancies were included in this study. Pregnancies with unknown delivery modes and non-singleton pregnancies were excluded from the analysis. Only the second pregnancy of the mother after CS as the first pregnancy was included, as the later pregnancies have too many uncontrollable factors, making the study design too unclear. Since cases of CS were classified as elective or urgent prior to 2004, we have used the same classification in the present study instead of the newer three-stage classification (elective, urgent, and emergency). This means that urgent and emergency CS is considered unplanned CS in this study. TOLAC was defined as all the other delivery modes, but elective CS, meaning that TOLACs include VBAC and unplanned CS. Delivery modes were defined according to the actual mode of delivery, meaning that patients who had planned elective CS before undergoing unplanned CS, were identified as unplanned CS.

**Fig. 1:**
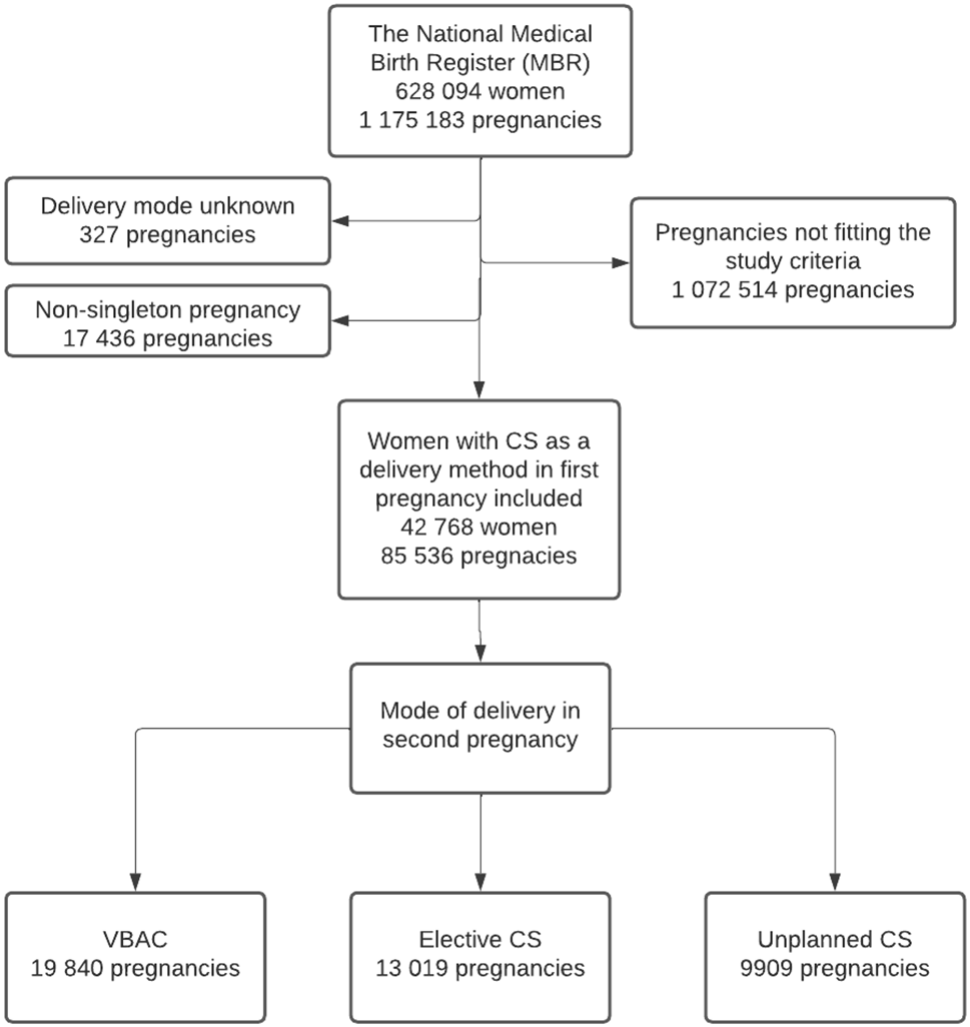
Flowchart depicting the process used to divide the study population into groups

A total of 42,768 filled the criteria to be included in this study. Of these, a total of 13,253 women had elective CS in the first pregnancy and 29,515 had unplanned CS in the first pregnancy. The process used to form the study groups is shown as a flowchart in Fig. 1.

### Statistics

Continuous variables were interpreted as means with standard deviations (SDs) or as a median with an interquartile range (IQR) based on the distribution of the data. The categorical variables are presented as absolute numbers and percentages. Absolute annual numbers and incidence rates for TOLACs and elective CS were calculated. In addition, TOLACS were still divided into VBAC and unplanned CS, and absolute numbers and incidence rates for these were calculated. The results of this study are reported according to STROBE guidelines [11].

### Ethics

Both the National Medical Birth Register (MBR) and the Care Register for Healthcare had the same unique pseudonymized identification number for each patient. The pseudonymization was made by the Finnish data authority FINDATA, and the authors did not have access to the pseudonymization key, as it is maintained by FINDATA. In accordance with Finnish legislation, no informed written consent was required because of the retrospective register-based study design and because the patients were not contacted. Permission for the use of this data was granted by FINDATA after evaluation of the study protocol (Permission number: THL/1756/14.02.00/2020).

## Results

The mean age at the first pregnancy (CS) was 28.9 years and 32.2 during the second pregnancy. The mean time difference between the first and second pregnancies was 3.3 years. The total rate for VBAC was 46.4%. The rate for second CS electively was 30.4%, and for TOLAC, turning to unplanned CS was 23.2%. Elective CS was more common after the first pregnancy being elective CS when compared to unplanned CS as the first mode of delivery. 42.1% vs 25.2% (Table 1).

**Table 1:** Background information on the study population, and absolute numbers and percentages of different delivery methods in total, and separately after a specific type of cesarean section (CS) (elective/unplanned) as the first mode of delivery

Total number of women included	42,768 women	
Age at the time of first pregnancy (mean; sd)	28.9 (4.7)	
Age at the time of second pregnancy (mean; sd)	32.2 (4.7)	
Time difference between pregnancies in years (mean; sd)	3.3 (2.0)	

	*n*	%
Delivery methods in 2nd pregnancy		
VBAC	19,840	46.4
Elective CS	13,019	30.4
Unplanned CS	9909	23.2
Total number of elective CS as first pregnancy	13,253	31.0
Mode of delivery after elective CS		
VBAC	5553	41.9
Elective CS	5582	42.1
Unplanned CS	2118	16.0
Total number of unplanned CS as first pregnancy	29,515	69.0
Mode of delivery after unplanned CS		
VBAC	14,287	48.4
Elective CS	7437	25.2
Unplanned CS	7791	26.4

*VBAC* vaginal birth after cesarean section

**Fig. 2:**
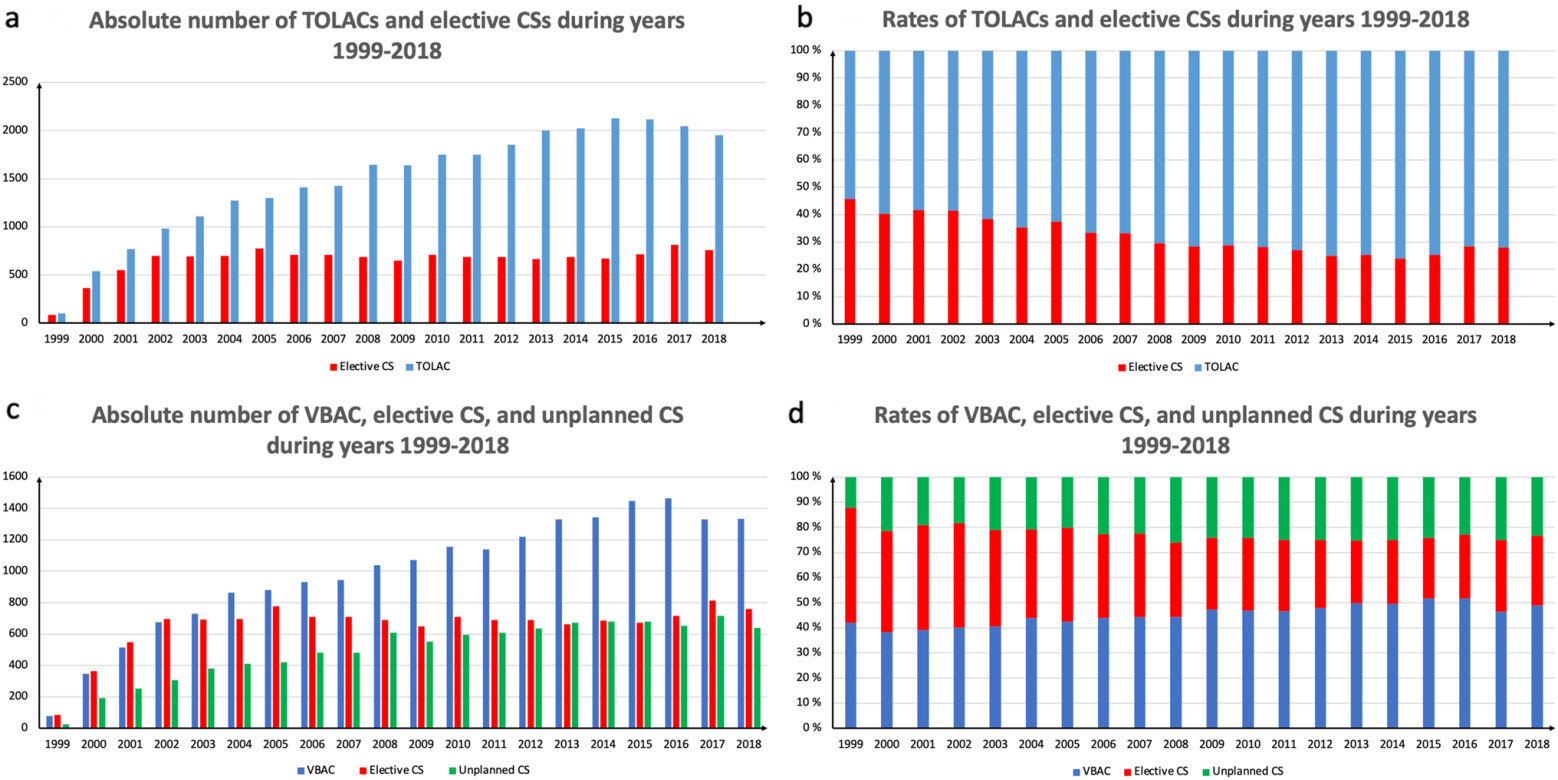
**a** Absolute number of trials of labor after cesarean sections (TOLACs) and elective cesarean sections (CS). **b** The annual rates of TOLACS and elective CS. **c** Absolute number of vaginal births after cesarean (VBACs), elective CS, and unplanned CS. **d** The rates of VBACs, elective CS, and unplanned CS

The total number of second deliveries with preceding CS increased strongly during the study period, indicating that the incidence of CS increased in nulliparous women. The absolute number of TOLACs had an increasing trend during our study period, increasing up to 2118 TOLACs in 2016. The absolute number of elective CSs peaked in 2016 but remained relatively stable during our study period. (Fig. 2a) The incidence rates for elective CS after the first CS had a decreasing trend, decreasing from 45% in 1999, to 28% in 2018. On the contrary, the rates for TOLACs increased from 55% in 1999 to 62% in 2018. (Fig. 2b) The absolute number of VBACs had an increasing trend during our study period, peaking in 2016 (1466 VBACs). (Fig. 2c) The rates for VBAC remained relatively constant, ranging between 38 and 52%, but had a slightly increasing trend towards the end of study period. (Fig. 2d).

## Discussion

The main finding of this study was that despite the increasing total number of deliveries with preceding CS in the first pregnancy, the absolute numbers and rates for VBACs increased during last decades towards the end of the study period in Finland. According to THL and previous literature [5, 12], the overall rates for CS remained relatively similar, but slightly increased during last decades in Finland. Interestingly, despite the decreased birth rate during the study period in Finland, [13] the absolute numbers of second deliveries with preceding CS increased, and the rate for repeat CS decreased.

According to a large nationwide study in China, the incidence of VBACs has also increased during the last decade [4]. However, it should be noted, that women in Finland who undergo delivery by CS are recommended to take 6–12 months to recover between the CS procedure and subsequent pregnancy, which partly explains the lower absolute numbers of deliveries during the first years of our study period, as women didn’t have time to get pregnant again. According to the previous literature, the median interval between the birth of the first child and the beginning of the next pregnancy was 20 months for the CS group and 18 months for the reference group, comprising vaginal deliveries [14]. The epidemiology of TOLACs and VBACs should be better investigated around the world, as with the rapidly increasing trend of CSs, these events are becoming a more common challenge in health care and therefore more information on these should be gained.

The total rate for VBAC during our study period was 49%. However, among TOLACs, the rate for VBAC was 67%, which is a better indicator for the success rate for VBAC, than the overall VBAC rate. The success rates of VBACs around the world have differed. In the United States, the success rate was found to be between 39 and 70% [7]. In Taiwan, the success rate of VBAC among mothers attempting vaginal delivery was 85% [8]. According to a large multicenter study in 2012, the total success rate of VBAC was 72% [15]. However, in this study, only the first VBACs were included, which most likely decreases the vaginal birth rate, as later VBACs, after a successful VBAC are known to have a higher success rate [15]. In addition, the success rates for VBACs increased slightly towards the end of the study period, indicating that some improvements may have occurred in obstetric care during the last decades.

In Finland, the decision of TOLAC is based on international recommendations [16, 17]. Although TOLAC is appropriate for many women, several factors increase the likelihood of a failed trial of labor, which in turn is associated with increased maternal and perinatal morbidity [17]. Therefore, assessing the likelihood of VBAC as well as the individual risks is important when determining who is an appropriate candidate for TOLAC [17]. According to a study in the United States, the rates of TOLAC have decreased due to fear of uterine rupture and medicolegal issues in there [7]. In Finland however, such a decrease in the rates of TOLACs is not observed.

The rates for elective CS after the first CS had a decreasing trend during our study period, which is an important finding. Also, the fact that repeat elective CS was much more common after elective CS, indicates that women willing to give birth by CS electively wanted to do so in their second pregnancy. This could be due to contraindications to deliver vaginally or psychologic factors, such as fear of childbirth. Thus, the prevention of the first CS is crucial. However, it appears that women having a trial of labour in their first pregnancy didn’t develop an insurmountable obstacle for the TOLAC, despite the preceding unplanned CS. According to previous literature, the main indications for elective CS in Finland were breech presentation, fear of childbirth, and fetopelvic disproportion [18]. According to a previous Finnish study, one of the strongest risk factors for fear of childbirth was previous CS [19]. Therefore, one part of the decreased rate of repeat CS might be the improvements behind the treatment of psychological factors such as maternal fear of childbirth. However, due to the crude nature of our data, the exact reason behind the decreased repeat CS rate remains unknown. The results of this should provide basic information on the epidemiology of TOLACs and VBACs, and further research should be made worldwide on the outcomes and success rates of these events using larger and more precise data.

The strengths of our study are the large nationwide register data used and the long study period, which allowed us to analyze the VBACs using a large study population. Register data used in our study are routinely collected in structured forms using national instructions, which ensures good coverage (over 99%) and reduces possible reporting and selection biases.

The main limitation of this study is that the indications behind CS delivery are not registered in the MBR, which means that indications for these delivery methods remain unknown. The initial intended mode of delivery is not registered in the MBR. Thus, some women in the TOLAC group may have had an elective CS scheduled, but it was registered as unplanned CS due to preterm onset of labor. However, in relation to all deliveries included in this study, the proportion of these is most likely relatively low and doesn’t, therefore, have important effects on our results. Furthermore, since cases of CS were classified as elective or unplanned prior to 2004, we have used the same classification in the present study instead of the newer three-stage classification (elective, urgent, and emergency).

## Conclusion

The main finding of this study was that despite the increasing annual total number of deliveries with CS in the first pregnancy, the absolute numbers and rates for VBACs have increased towards the end of the study period in Finland. The epidemiology of TOLACs and VBACs should be better studied around the world, as with the rapidly increasing rate of CSs, these events are becoming more common challenges in health care.

## Data Availability

Data cannot be shared publicly because of Finnish reglulations. There are legal restrictions on sharing data publicly. Data is not available without the permission from the Finnish authority Findata (Url Findata.fi). Permission to use the data was granted by Findata after evaluation of the study protocol (Permission number: THL/1756/14.02.00/2020). For researchers who meet the criteria for access to confidential data, Findata can be contacted via email (info@findata.fi).
